# vSPACE: exploring virtual spatial representation of articular chondrocytes at the single-cell level

**DOI:** 10.1093/bioinformatics/btae568

**Published:** 2024-10-03

**Authors:** Chenyu Zhang, Honglin Wang, Yeonsoo Chung, Seung-Hyun Hong, Merissa Olmer, Hannah Swahn, Martin Lotz, Peter Maye, David Rowe, Dong-Guk Shin

**Affiliations:** School of Computing, University of Connecticut, Storrs, CT 06269-4155, United States; School of Computing, University of Connecticut, Storrs, CT 06269-4155, United States; School of Computing, University of Connecticut, Storrs, CT 06269-4155, United States; School of Computing, University of Connecticut, Storrs, CT 06269-4155, United States; Departments of Molecular and Cell Biology and Molecular Medicine, Scripps Research, La Jolla, CA 92037, United States; Departments of Molecular and Cell Biology and Molecular Medicine, Scripps Research, La Jolla, CA 92037, United States; Departments of Molecular and Cell Biology and Molecular Medicine, Scripps Research, La Jolla, CA 92037, United States; Department of Reconstructive Sciences, UConn Health, Farmington, CT 06030-3705, United States; Center for Regenerative Medicine and Skeletal Development, UConn Health, Farmington, CT 06030-3705, United States; School of Computing, University of Connecticut, Storrs, CT 06269-4155, United States

## Abstract

**Summary:**

vSPACE is a web-based application presenting a spatial representation of scRNAseq data obtained from human articular cartilage by emulating the concept of spatial transcriptomics technology, but virtually. This virtual 2D plot presentation of human articular cartage cells generates several zonal distribution patterns, for one or multiple genes at a time, revealing patterns that scientists can appreciate as imputed spatial distribution patterns along the zonal axis.

**Availability and implementation:**

vSPACE is implemented in Python Dash as a web-based toolbox designed for data visualization of zonal gene expression patterns in articular cartilage chondrocytes. This tool is freely accessible at: https://vspace.cse.uconn.edu/

The source code and extra materials for this service can be downloaded from: https://github.com/zhacheny/vSPACE

## 1 Introduction

**Figure 1.**
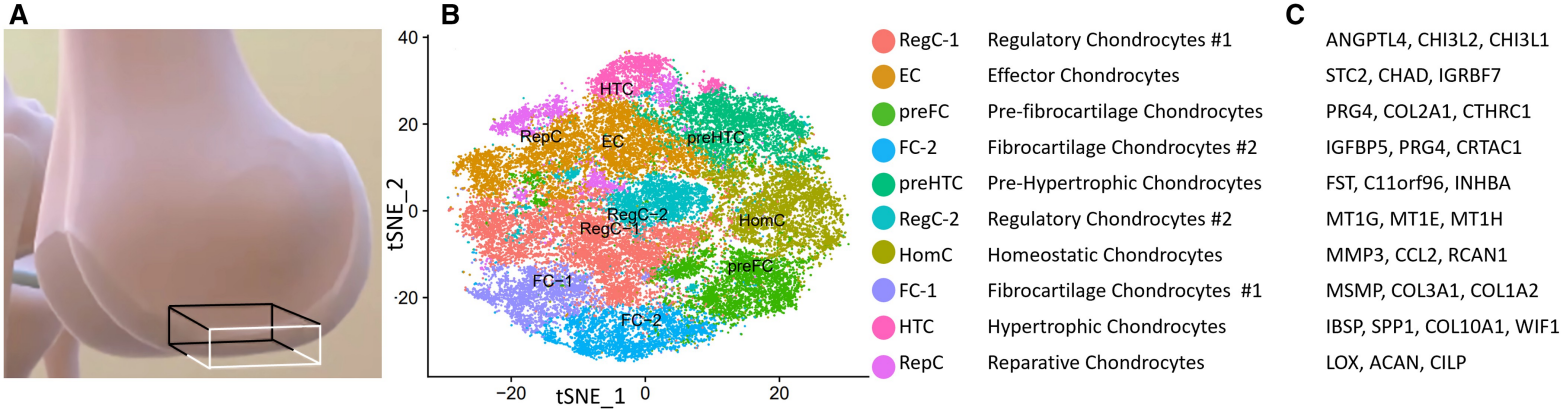
(A) Normal human femoral epiphyseal articular cartage biopsy region from which scRNA-seq data was obtained. (B) tSNE graphics of the named clusters from the study (Swahn *et al.* 2023). (C) The list of key genes used to produce the cluster names—inconsistent with known biology of this tissue.

Single cell and single nuclear RNA sequencing (scRNAseq and snRNAseq) is fast becoming the transcriptional standard for assessing the molecular landscape of a tissue because it reveals the cellular heterogeneity of the tissue that is lost with bulk RNAseq. The huge datasets resulting from these studies (5–10K cells by 10–20K genes) present a major challenge for the graphical representation of the types of cells with a similar molecular signature that are imputed into functional properties. In tissues where there are well-defined molecular signatures (e.g. the immune system), clustering cells into distinct and biological subgroups using widely used Seurat software (Butler *et al.* 2018) and tSNE/UMAP visualization is distinctive and biologically meaningful. However, in tissues where cell types are not well defined at the molecular level because prior knowledge is not well developed, such as articular cartilage (AC), the outcome generates clusters of merged cell populations with nomenclature that is not meaningful to the chondrocyte biological community. Figure 1 illustrates the tSNE analysis developed by our group using the Seurat program that identifies clusters with names of fibrocartilage, reparative, regulatory, homeostatic, and effector chondrocytes that do not align with the known properties of this tissue. Using UMAP also produces a very similar highly integrated one clump of cells that are not separated in the 2D display (Swahn *et al.* 2023). The tSNE/UMAP approach often misidentifies functional clusters because unbiased selection of the most variable transcripts fails to capture functionally important genes in part because the transcript changes of functionally discerning genes are subtle (not as dramatic as cell type discerning marker genes). Figure 1B is a classic example illustrating the limitation of using tSNE/UMAP style method when the gene expression values are obtained from one homogeneous cell type such as chondrocytes as in the case of AC.

**Figure 2.**
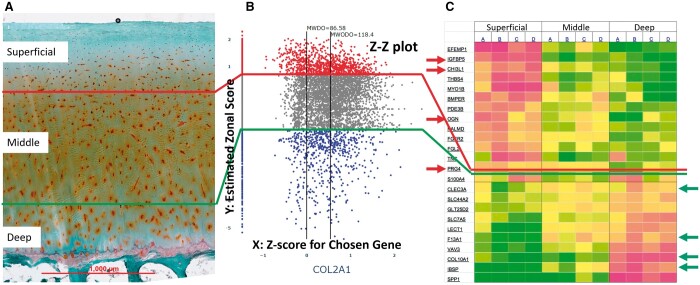
(A) Three zones of AC are identified from microscopic tissue histological image. (B) Z-Z plot emulates placement of cells by estimating zonal scores for individual cells for *Y*-axis and using expression *z*-score for the chosen gene (COL2A1) for *X*-axis. The vertical dots on the *Y*-axis are cells that do not express COL2A1. (C) Zonal marker genes identified for superficial and deep zones from gene expression study (Grogan *et al.* 2013) presented in the heat map are used to position each cell onto the Z-Z plot.

Here, we report on a graphical approach vSPACE (virtual Spatial Articular Cartilage Explorer) for representing a scRNAseq study of a tissue with well described histological organization, but which lacks the detailed cellular knowledge required for biologically meaningful clusters. It is designed to assist the AC community to develop meaningful clusters that eventually would be useful for spatial location of the clusters with the AC tissue. It is based on well-defined histological zone in the AC (superficial, transitional, middle and deep; Fig. 2A) that have been further refined at the transcriptional level by bulk RNAseq analysis of serial sections from the superficial to deep zone (Fig. 2C).

vSPACE differs from the recent multi-omics analysis systems that map scRNAseq data to spatial transcriptomics data (ST) data such as CytoSPACE (Vahid *et al.* 2023), CellTrek (Wei *et al.* 2022) and SpaOTsc (Cang and Nie 2020). These latter systems assume the availability of ST data (e.g. 10× Visium) and attempt to map disassociated scRNAseq data to the signals obtained from pockets of spatially anchored bulk cells, whereas vSPACE assumes no use of ST data.

## 2 Graphical design concept

Positioning each cell from scRNAseq study of AC within a 2D space (Fig. 2C) is based on two determinants. The *Y*-axis represents the estimate of the tissue’s top-to-bottom spatial positioning, meaning middle zone spreads out equidistant from *Y*’s value zero and *Y*’s higher positive value and lower negative value are mapped to, respectively, toward superficial and deeper zones. The *Y* value for each plotted cell reflects its similarity of gene expression to signal strength of the superficial or deep Zone Marker Genes (ZMG) that were selected from serial-section bulk RNA study as shown by the arrows in Fig. 2C (Grogan *et al.* 2013; PMID 23124445). The calculation of *Y* for each cell in the study is derived from the difference of the weighted summed superficial ZMG score minus the weighted summed deep ZMG score. For the *X*-axis the value represents the concerning gene expression strength in term of *z*-score, i.e. *X*-axis value 0 represents the gene’s population mean expression value and the *X* value being negative or positive means, respectively, expression value being smaller or larger than the population average.

## 3 Implementation

The developed vSPACE is a web-based toolbox for data processing, exploration, analysis and visualization of zonal gene expression patterns in AC cells. This web-based tool is written in the Python programming language (version ≥3.6.0) and takes advantage of the Dash (Dabbas 2021) for the pipelined and customizable visualization to produce publication-ready figures and tables.

### 3.1 Input data

The input dataset needs to be preprocessed before being fed into the vSPACE portal. This step converts the raw dataset into *z*-score format and computes all data matrices for a single cell including the averages of the gene expression values for each cell with the dropouts (genes that are not expressing), the averages of the gene expression value for each cell without the dropouts and the zonal labels. After this step, the processed data format also stores all the metadata, including the cell barcodes, gene symbol names, lists of marker gene sets, tissue types as well as the zonal names. Upon data input, a virtual spatial representation of the data points is then displayed in the interactive viewer in 2D space, also known as the “Z-Z plot” (Zone versus *Z* score). The following parts illustrate how the Z-Z plot is constructed.

### 3.2 Computing zonal score

Spatial markers for the AC cells are assumed to be known a priori, which we call the ZMG. How to find the most ideal ZMG for a single cell study performed on AC cells is an on-going research topic. In this study, we use the zonal markers produced by serial sections from the superficial to the deep zones analyzed with Mass Spec (Müller *et al.* 2014; PMID 25193283) and bulk RNAseq (Grogan *et al.* 2013; PMID 23124445) to compile a list of ZMG in the two major zones (superficial and deep) of the AC. Zonal scores are calculated by Equation (1). We note that the formula given below is a special form of a similar equation that has been already detailed in our previous work (Zhang *et al.* 2022).

(1) $$\begin{array}{c} Z_{c}=\sum_{g\;\in \;ZMG_{S}}E_{g,c}/\left|ZMG_{S}\right|-\sum_{g\;\in \;{\mathrm{ZMG}}_{D}}E_{g,c}/\left|{\mathrm{ZMG}}_{D}\right| \end{array}$$

where $E_{g,c}$ is the *z* score expression value of the gene *g* of the cell *c*, $ZMG_{S}$ and $ZMG_{D}$ represent, respectively, the superficial zone markers and deep zone markers. The notations $\left|ZMG_{S}\right|$ and $\left|ZMG_{D}\right|$ correspond to the number of the marker genes in the superficial zone and the deep zone, respectively. $Z_{c}$ is calculated after trimmed quantile normalization is performed on the raw unique molecular identifier (UMI) count values (Wang *et al.* 2022). Figure 2B plot is generated using PRG4, IGFBP5, CHI3L1, and OGN for $ZMG_{S}\;$and COL10A1, integrin binding sialoprotein (IBSP), CLEC3A and F13A1 for $ZMG_{D}$ indicated by arrows in Fig. 2C.

### 3.3 Developing/validating the computational spatial map

For its 2D placement of single cells, the Z-Z plot places each AC cell on the *Y*-axis into three zone—superficial zone, middle zone and deep zone—based the similarity of the expression profile to the serial bulk RNAseq where the *z* score for all cells expressing the gene determines the *Y*-axis value (Fig. 2B). Note that cells not expressing the gene (dropouts) are placed on the *Y*-axis line. As shown in Fig. 2B, the Z-Z plot includes two horizontal lines, the upper one distinguishing superficial zone and middle zone and the lower one distinguishing middle zone and deep zone. The data points are colored by the zonal scores: cells (i.e. dots) in superficial zone are red colored and cells in deep zone are colored blue. The middle zone cells are colored gray. Here the position in *Y*-axis conveys the likelihood of a cell’s zonal membership. For example, the topmost positioned cells are estimated to be the most likely superficial zone cells and the bottommost positioned ones most likely deep zone cells. Cells are positioned along the *Y*-axis based on the corresponding zonal scores estimating where they should be between the two extremes. The plot also includes two vertical lines, the left one identifying the average gene expression value with dropouts (left vertical line) and the right vertical one the average value of expressing cells (dropouts excluded) for the selected gene whose symbol placed below the *X*-axis. The two vertical lines are annotated with numeric values denoting the average intensity values of the data points that are generated for the chosen gene.

**Figure 3.**
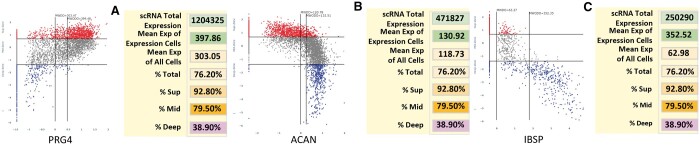
Z-Z plot of three genes whose distribution is well known by the AC community. (A) Lubricin (PRG4) is produced by superficial zone cells. (B) Aggrecan (ACAN) is a major matrix proteoglycan primarily located in the mid and deep zone. (C) Integrin binding sialoprotein (IBSP) is a major matrix protein of the deep zone. For each graph, the zonal dot percentage table shows mean level of total and per cell expression and percent cellular distribution within each histological zone.

The program initially generates a Zonal Dot Count Table for the nine Z-Z compartments (non-expressing, Z < 0, Z > 0 in the superficial, middle and deep zone). Subsequently it produces the Zonal Dot Percentage Table (Fig. 3) that calculates percentage of all cells expressing the gene in each zone, excluding dropouts as well as the summed expression of the gene in all the cells in the scRNAseq study and the mean expression per total cell number and per expressing cells (non-expressing cells removed).

**Figure 4.**
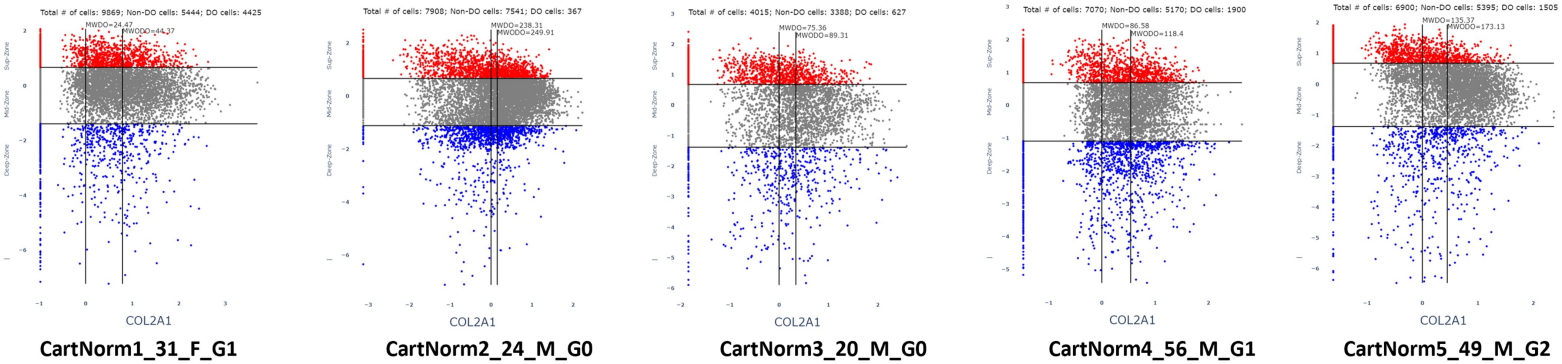
Consistency of the Z-Z plot for COL2A1 across AC samples obtained from health donors. Not shown the comparison of the total and per cell expression across the donor set that does seem to vary by sex and donor age.

Distinctive Z-Z patterns are produced when cells are selected for genes expressed in the three distinct zones. PRG4 produces the lubricin protein that is synthesized in the superficial zone and secreted into the joint fluid. The Z-Z pattern shows strong expression in the superficial and upper middle zone (Fig. 3A). Aggrecan (ACAN) is a major proteoglycan that accumulates in the middle and deep zones, which is reflected in the Z-Z pattern of strong expression in the two lower zones (Fig. 3B). IBSP is a characteristic protein of the deep zone, and the Z-Z pattern matches this expectation by strong expression in the deep zone only (Fig. 3C). Another validating observation is the Z-Z pattern of cell distribution of a single gene across multiple human donors selected by not having evidence of disease or trauma. Figure 4 illustrates the example of cells expressing the COL2A1 gene across five human subjects (one female, four male) ranging in age from 20 to 56. While the patterns are the same, the mean level and percent of expression can vary and could be an early marker of a subtle perturbation. Additional Z-Z patterns are provided in Supplementary Fig. S1 with accompanying explanations.

### 3.4 Use of spatial mapping to discover clusters

**Figure 5.**
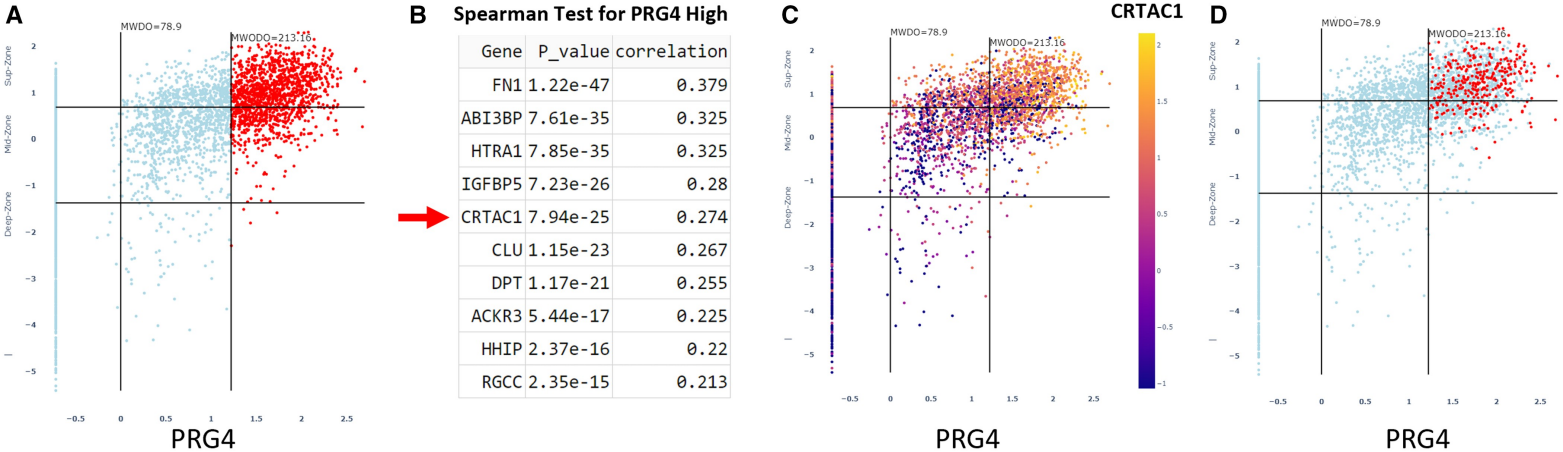
Graphical representation of cell clusters based on co-expression of marker genes. (A) Z-Z plot of PRG4. (B) Spearman test for PRG4 high cells. CRTAC1 emerges. (C) Heat map of co-expressing CRTAC1 cells overlayed onto the PRG4 pattern showing high expression (*Z* > 1) of both genes in the superficial zone. (D) Overlay of cells with strong expressing (*Z* > 1) of three genes (CRTAC1, DPT, and ABI3BP) in PRG4 positive cells that show localization to the superficial zone.

Although the cellular histology of each zone has a similar morphology, vSPACE reveals a very heterogeneous population when viewed as molecular clusters. The analysis begins by selecting a well understood gene that is expressed in subpopulation of the AC such as PRG4, ACAN, COL2A1, and IBSP (what we call “cluster sentinel genes”). Using the available Spearman test, the genes most frequently co-expressed with the PRG4 sentinel gene are discoverable. Figure 5 shows how the discovered co-expressing gene CRTAC1 (cartilage acidic protein) can be examined. Figure 5A shows the Z-Z pattern of PRG4 only (single gene mode). Figure 5B shows the outcome from Spearman test. Figure 5C overlays a heat map expression of CRTAC1 onto the PRG4 cells (in a co-expressing mode). Cells that strongly express both PRG4 and CRTAC1 have the bright orange color and are located in the superficial and top portion of the middle zone. Cells that are colored blue show low or no expression of both genes and are located in the middle to deep zone. The Zonal Dot Count and Percent program then generates the total number of transcripts, mean values of expressing cells and the percent enrichment within the cluster relative to the total cell population within each histological zone.

The highly co-expressing cells (orange-colored cells) in the superficial zone can be exported into an output file to tabulate other genes that are expressed within the two-member cluster using Multiple Genes selection feature available in the program. Using the feature, other highly expressed gene(s) can be selected to further test the specificity of the cluster. Figure 5D illustrates a multigene-co-expressed cluster, i.e. highly co-expressing PRG4-CRTAC1-DPT (dermatopontin, a secreted integrin binding protein) and ABI3BP (a secreted adaptor SH3 protein). The red dots within the entire PRG4 pattern are the cells that express the 4-member cluster. The user can perform another round of exploration in real-time, i.e. Spearman correlation test to find other genes enriched in the cluster.

### 3.5 Use by the AC biomedical community

The program is available to the public as a web-based tool at https://vspace.cse.uconn.edu. Currently there are 13 scRNAseq datasets preloaded by processing publicly available human AC scRNAseq datasets. Examining each of these datasets can begin by simply choosing a dataset from the dropdown menu listing the choices. The preloaded datasets include six human knee healthy AC samples and six human knee osteoarthritis (OA) samples (GSE220243) and one pooled dataset from 34 human OA samples (GSE104782). This computational environment offers two primary analytic capabilities that will enhance the understanding of scRNAseq experiment of AC. The first analytic capability is to review the cellular distribution patterns of one or more selected genes over the Z-Z plot (explained in Section 3.3) with “Use Gradient Color” option. One may enter FGFBP2 in the primary query box of the portal and discover its exceptionally formed negative correlation pattern along the zonal axis (Supplementary Fig. S1). This gene has been annotated for only a very broad GO Biological Process term “cell-cell signaling” (GO:0007267) suggesting that its function is not well studied in general, not to mention its regulatory role in AC chondrocytes. Since the pattern of ACAN resembles that of FGFBP2 (Supplementary Fig. S1), this scientist can explore how the two genes overlap by entering ACAN in the secondary query box and will discover an extreme consistency both in expression level and localization between the two gene through heatmap visualization (e.g. Fig. 5C). The second analytic capability is to further drill down this “unusual” finding using the option “Use Cell Section” (explained in Section 3.4). The scientist selects a cluster of cells that are highly expressed both in FGFBP2 and ACAN and performs Spearman test to find which other gene(s) may be highly co-expressed in this subgroup. The secondary query box can include multiple genes to help further refine this cell cluster discovery effort. These capabilities are designed to develop meaningful clusters and analytical pathways to supplant the current tSNE/UMAP approach. The response rate of the portal is dependent on the size of the dataset (e.g. 7071 cells × 445 genes versus 4450 genes) which determines the complexity of the clusters that can be assessed with the tool.

## 4 Conclusions

Our in-house use of the program environment has led us to appreciate the cellular complexity of a tissue that histologically appears to be very homogeneous. For example, a cluster based on strong COL2A1-COL3A1 co-expression reveals a cell population specializing in synthesis of multiple collagen types (COL5, COL6, COL9, COL11, COL12, COL15, COL16, COL27) while a COL2A-CTHRC1 cluster makes no collagen transcripts but instead secretes matrix and growth factor modifying products. The Z-Z plot dramatically convey the difference between the pattern of COL2A1 between healthy and osteoarthritic cartilage with the percentage of cells expressing the transcript increasing from 60% to 100% (although a lower total number of cells and the per cell expression increasing from 80 to 240). While bulk RNA sequencing does show a diminished COL2A1 mRNA, the scRNAseq provides a totally different perspective of how the AC chondrocyte responds to the disease environment. We predict that even more sophisticated examples of early and late-stage disease will become evident when biological clusters are utilized for further interrogation. Finally, as marker gene combinations are discovered and made public, it will provide guidance for probe selection when designing and interpreting cell level spatial transcriptomic or proteomic studies. This web portal is designed to be a community resource—a knowledge portal for the AC research community.

## Supplementary Material

btae568_Supplementary_Data

## Data Availability

All the example data and analytic code for this web service are available at: https://github.com/zhacheny/vSPACE.

## References

[btae568-B1] Butler A , Hoffman P, Smibert P et al Integrating single-cell transcriptomic data across different conditions, technologies, and species. Nat Biotechnol 2018;36:411–20. 29608179 10.1038/nbt.4096 PMC6700744

[btae568-B2] Cang Z , Nie Q. Inferring spatial and signaling relationships between cells from single cell transcriptomic data. Nat Commun 2020;11:2084. 32350282 10.1038/s41467-020-15968-5 PMC7190659

[btae568-B3] Dabbas E. Interactive Dashboards and Data Apps with Plotly and Dash: Harness the Power of a Fully Fledged Frontend Web Framework in Python–No JavaScript Required. Birmingham, England: Packt Publishing Ltd, 2021.

[btae568-B4] Grogan SP , Duffy SF, Pauli C et al Zone-specific gene expression patterns in articular cartilage. Arthritis Rheum 2013;65:418–28. 23124445 10.1002/art.37760 PMC3558601

[btae568-B5] Müller C , Khabut A, Dudhia J et al Quantitative proteomics at different depths in human articular cartilage reveals unique patterns of protein distribution. Matrix Biol 2014;40:34–45. 25193283 10.1016/j.matbio.2014.08.013

[btae568-B6] Swahn H , Li K, Duffy T et al Senescent cell population with ZEB1 transcription factor as its main regulator promotes osteoarthritis in cartilage and meniscus. Ann Rheum Dis 2023;82:403–15. 36564153 10.1136/ard-2022-223227 PMC10076001

[btae568-B7] Vahid MR , Brown EL, Steen CB et al High-resolution alignment of single-cell and spatial transcriptomes with CytoSPACE. Nat Biotechnol 2023;41:1543–8. 36879008 10.1038/s41587-023-01697-9 PMC10635828

[btae568-B8] Wang H , Joshi P, Zhang C et al rCom: a route-based framework inferring cell type communication and regulatory network using single cell data. In: *Proceedings of the 13th ACM-BCB*, Northbrook, IL. New York, NY: Association for Computing Machinery, 2022, 1 5.

[btae568-B9] Wei R , He S, Bai S et al Spatial charting of single-cell transcriptomes in tissues. Nat Biotechnol 2022;40:1190–9. 35314812 10.1038/s41587-022-01233-1 PMC9673606

[btae568-B10] Zhang C , Joshi P, Wang H et al Pola Viz reveals microglia polarization at single cell level in Alzheimer’s disease. In: 2022 IEEE Inter*national* Conf*erence* on Bioinfo*rmatics* and Biomed*icine (BIBM)*, Las Vegas, NV. Piscataway, NJ: Institute of Electrical and Electronics Engineers (IEEE), 2022, 1387–92.

