# Supplementary material for: vSPACE: exploring virtual spatial representation of articular chondrocytes at the single-cell level
Source: Bioinformatics. 2024 Oct 3;40(10):btae568. doi: 10.1093/bioinformatics/btae568 (PMC11483105; doi:10.1093/bioinformatics/btae568)
Supplement: btae568_Supplementary_Data [file btae568_supplementary_data.pdf]

Supplementary Information

vSPACE: Exploring Virtual Spatial Representation of Articular Chondrocytes at the Single-Cell Level

Gene	Fig. S1	Spearman_corr	p_value	Pearson_corr	p_value	Spearman	Pearson	Marker
OGN	A	0.64795334	0	0.594735155	0	**		Yes
PRG4	B	0.60486126	0	0.53063477	0	**		Yes
CRTAC1	C	0.527483648	0	0.46498667	0	**		
CHI3L1		0.509971036	0	0.532882724	0		**	Yes
IGFBP5	D	0.50607466	0	0.440195273	0	**		Yes
ABI3BP	E	0.302244078	2.93E-149	0.24907047	1.93E-100	**		
TPT1	F	0.227611951	9.37E-84	0.134245801	8.59E-30	**		
FN1		0.030770002	0.00967	-0.049847901	2.75E-05		**	
COL2A1	G	-0.165246756	1.84E-44	-0.145941762	5.86E-35	**		
SPP1		-0.358299288	3.48E-213	-0.556724981	0		**	
COL10A1	H	-0.367102427	1.76E-224	-0.595645899	0		**	Yes
IBSP	I	-0.401728334	1.75E-272	-0.621393021	0		**	Yes
FRZB		-0.552933145	0	-0.370712515	3.23E-229	**		
ACAN	J	-0.624052524	0	-0.4749436	0	**		
CLEC3A	K	-0.659504426	0	-0.603158643	0	**		Yes
FGFBP2	L	-0.762702831	0	-0.658220979	0	**		

Supplementary Table S1: This table is to contrast <correlation coefficient, p-value> from Pearson and Spearman tests for the X and Y values obtained for selectively chosen 16 genes using the entire population of cells (total number, 7068) from the human knee articular cartilage sample CartNorm4_56_M_G1 (56 years old, Male, Sample Grade 1). The objective of this table is to contrast linear or non-linear nature of "correlation" trends between the z-score for the chosen gene (X value) and the "surrogate" zonal score (Y value) for all cells. One question under consideration is if Equation 1 prescribes the outcome patterns to follow linearity exclusively or not. One way to test the linearity presumption by the equation is to perform two different correlation tests between actual X and Y values that are produced for the population of cells and compare the coefficient values from the two tests, Pearson measuring linear associating and Spearman measuring monotonic relationship. By comparing the coefficients from the two tests, one can gauge the likely linear/or non-linear trend inherently present in the population of the cells. For each row (gene) in the table, the larger (stronger) coefficient cases are denoted by **. Marker column indicates if the selected gene is a zonal marker gene used to calculate the zonal score or not. Absent value here means not a marker gene. The comparison shows that in 11 out of the total 16, the magnitude of Spearman coefficient values is larger. In contrast, in 5 out of 16 cases Pearson coefficient is larger indicating a higher degree of linearity. Noticeable here are the 3 out 5 these cases are for the marker genes which is expected due to the nature of algebraic formulation of Equation 1. Since these coefficients are only indicative of trend (positive value for positive correlation and negative value for negative trend), the actual Z-Z plots for a subset of these genes are presented in Supplementary Figure S1.

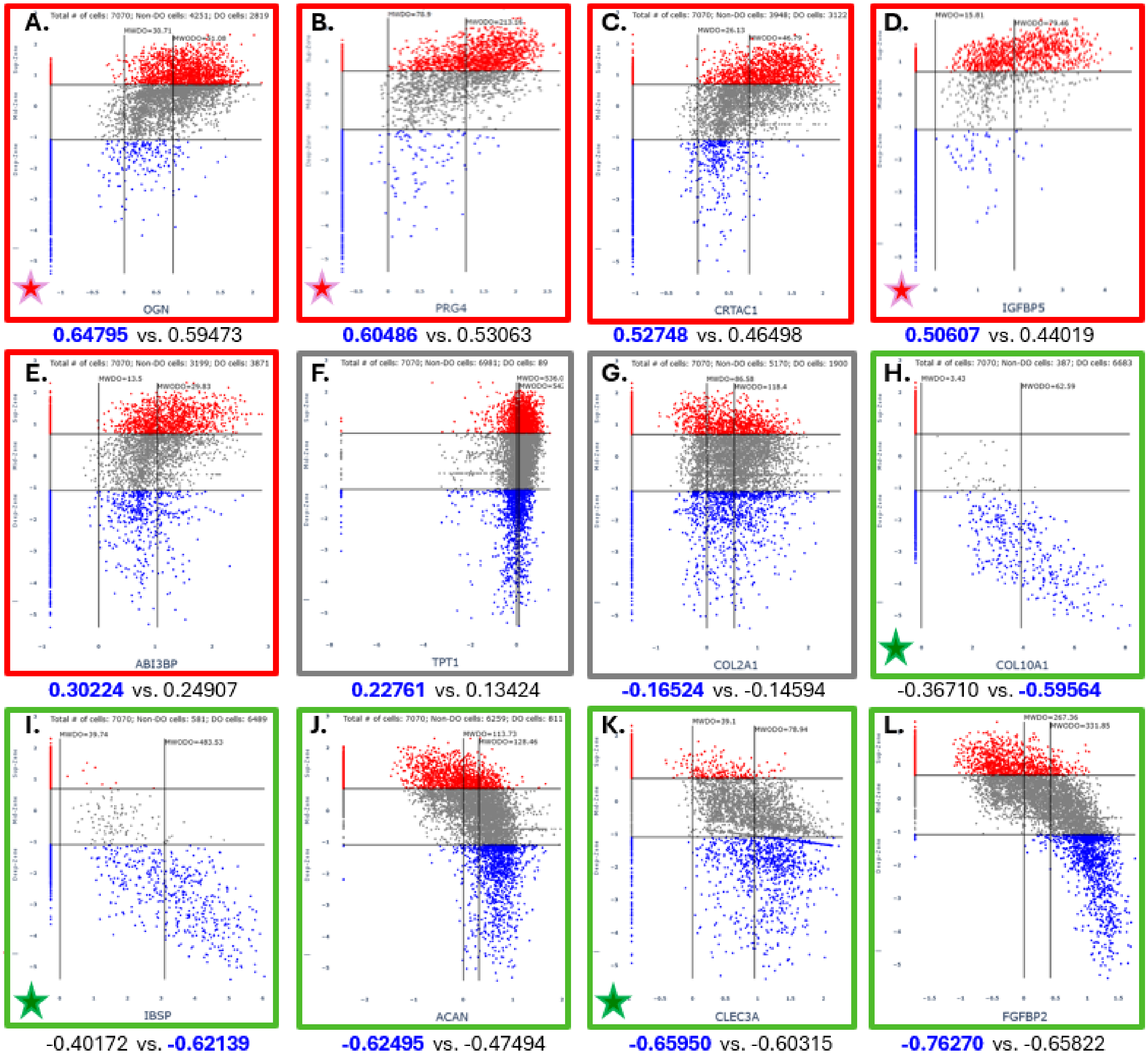

Supplementary Figure S1: Selective Z-Z plots from the genes in Supplementary Table S1 are presented to help the readers develop intuition in interpreting the reported correlation coefficients. Each plot is annotated with two numbers in the format, Spearman coefficient vs. Peason coefficient. The boldface blue colored number signifies a larger absolute value between the two. The figure IDs A-L match the IDs given in Fig. S1 column in Table S1 thus following the rank order of the genes listed based on the Spearman coefficients (from the highest to the lowest). Star icons are placed to indicate marker gene status, OGN (A), PRG4 (B) and IGFBP5 (D), for the superficial zone (with red color stars) and COL10A1 (H), IBSP (I) and CLEC3A (K) for the deep zone (with green color stars). The correlation trend is indicated by the plot outline color, Red for positive correlation and Green for negative correlation. Gray suggests little correlation such as for TPT1 (F) and COL2A1 (G). TPT1 is a great example for no correlation and this figure has been instrumental for biologists to decide to use TPT1 as a control in their spatial transcriptomics experiments since this gene should be highly expressing in all zones. A few notable facts in this figure are summarized. (i) The genes with higher Spearman coefficient than Pearson coefficient exhibit non-linearity trend as expected. (ii) There are genes exhibiting a linear trend as in COL10A1 (H) and IBSP (I) and this is not surprising because these two genes are marker genes used to produce Y axis values. (iii) However, being a marker gene does not mean the trend should be linear as evidenced by the patterns and the higher Spearman coefficients for other marker genes OGN (A), PRG4 (B), IGFBP5 (D), and CLEC3A (K). (iv) Lastly, strongly correlated genes are newly discovered such as CRTAC1 (C), ACAN (J) and FGFBP2 (L) which clearly show non-linear trends and strongly suggest spatial locality of gene expression patterns along the zonal axis. Such information has been very useful for bench scientists.

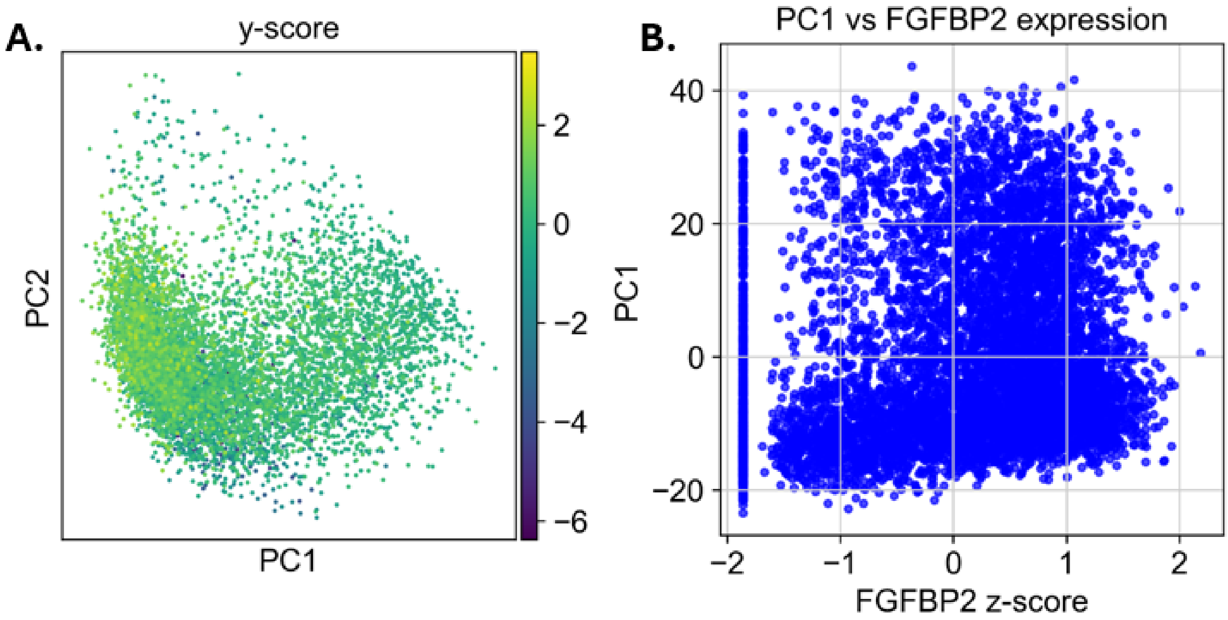

Supplementary Figure S2. PCA is an established, widely used dimension reduction method. Our Zonal scoring can be seen as a dimension reduction method, that is, mapping the gene expressions into one dimension, i.e. the Y axis in our Z-Z plot. One might be interested in examining how the Zonal score is compared with the principal components of the PCA analysis. Shown in (A) is overlaying each cell's Zonal score over the scatter plot in which cells are placed based on their two top PC components, i.e., using PC1 for the X axis and PC2 for the Y axis. The heatmap coloring for each cell is done using the Zonal score, i.e., y axis score in our Z-Z plot. The color pattern clearly suggests little association between the two top PC components and the Zonal scores. We also show a different but related analysis outcome in (B). Since the Z-Z plot is designed to examine each gene's z-score along the Y axis, in (B) we attempt to produce a scatter plot which places each cell based its z-score for the X axis and PC1 for the Y axis. Shown in (B) is using the z-scores for FGFBP2. Comparing this figure (B) with the pattern (L) in Figure S1 clearly demonstrates that PC1 may have little to do with the spatial information of articular cartilage. Use of PC2 produces a similar outcome—no pattern (not included here due to space limitation). Neither PC1 nor PC2 captures AC tissue's spatial relationship either individually or together. We note that FGFBP2 was not used in creating the Zonal score. It would be extremely unlikely that such a highly negatively correlated pattern between the Zonal score and the z-scores of FGFBP2 (shown in Figure S2 (L)) can appear "by chance".
